# Burden of renal angiomyolipomas associated with tuberous sclerosis complex: results of a patient and caregiver survey

**DOI:** 10.1186/s41687-018-0055-4

**Published:** 2018-07-13

**Authors:** Anne M. Rentz, Anne M. Skalicky, Zhimei Liu, David W. Dunn, Michael D. Frost, Jo Anne Nakagawa, Judith Prestifilippo, Qayyim Said, James W. Wheless

**Affiliations:** 1 Evidera, 7101 Wisconsin Avenue, Suite 1400, Bethesda, MD 20814 USA; 2 Novartis Oncology, East Hanover, NJ USA; 3 Riley Hospital for Children, Indianapolis, IN USA; 4 Minnesota Epilepsy Group, St. Paul, MN USA; 5 Tuberous Sclerosis Alliance, Silver Spring, MD USA; 6 Le Bonheur Children’s Hospital and the University of Tennessee, Memphis, TN USA

**Keywords:** Health care utilization, Health-related quality of life, Renal angiomyolipomas, Tuberous sclerosis complex (TSC)

## Abstract

**Background:**

Tuberous sclerosis complex (TSC) is a rare genetic disorder characterized by benign tumors in multiple organs, including non-cancerous kidney lesions known as renal angiomyolipomas. This study’s objective is to describe the age-stratified morbidity, treatment patterns, and health-related quality of life of TSC patients with renal angiomyolipomas in the United States. A cross-sectional, anonymous web-based survey was conducted with a convenience sample of TSC patients and caregivers identified through a patient advocacy organization.

**Results:**

Out of the total sample of 676, 182 respondents reported having kidney complications with 33% of the pediatric group and 25% of the adult group with TSC reporting them. Of those with kidney complications, 110 (60%) reported a diagnosis of renal angiomyolipomas, of which 79 (72%) were adult patients and 31 (28%) were pediatric age patients. Eighty-four percent of the pediatric group and 76% of the adult group reported lesions on both kidneys. Of the patients experiencing involvement of only one kidney, 60% of the pediatric group and 21% of the adult group reported having multiple tumors within the affected kidney. Almost all of the sample (99%) reported seeing a physician and having a procedure or test for TSC in the past year. Less than half the respondents (44%) reported being hospitalized in the past year. Thirty-nine percent reported an emergency room visit as well. Compared to scores for patients with kidney disease, the angiomyolipoma adult patients reported significantly lower Mental Component Summary scores on the SF-12.

**Conclusions:**

Renal angiomyolipomas burden leads to frequent healthcare resource use including hospitalization, invasive treatments, and surgical procedures, which result in an impaired mental health related quality of life.

## Background

Tuberous sclerosis complex (TSC) is a multisystem genetic disorder characterized by benign tumor growth in multiple vital organs, such as the brain, heart, skin, eyes, kidneys, and lungs, which can lead to a combination of symptoms or manifestations, including seizures, developmental delay, behavioral problems, skin abnormalities, and kidney disease [1, 2]. TSC is a rare disease with an incidence estimated at one in 6000 births [3, 4] and a prevalence of 1 in 9407 individuals; approximately 25,000–50,000 individuals with TSC live in the United States [2, 5, 6]. Neoplasia (pervasive epithelial cells forming vascular, smooth muscle and adipose tissue) seem to be responsible for many if not all of the tuberous sclerosis manifestations [7]. The severity of TSC can range from mild skin abnormalities to mental retardation or renal failure in severe cases.

Among TSC manifestations, kidney damage is the second cause of mortality/morbidity for all ages combined and the first cause of mortality after 30 years of age [8–12]. Renal manifestations are most commonly angiomyolipomas, but may be renal cysts, or in rare cases polycystic kidney disease or renal cell carcinoma. TSC angiomyolipomas are benign renal neoplasms composed of abnormal vessels, immature smooth muscle tissue, and adipocytes that can affect one or both kidneys [13, 14] with a prevalence between 34 and 80% in TSC patients [10, 15, 16]. There are over 10 million people world-wide with renal angiomyolipomata and approximately one tenth of these also have tuberous sclerosis complex [7]. Small angiomyolipomas that are not growing may not warrant treatment. However, morbidity for patients with TSC may be high in cases where angiomyolipomas are numerous and/or large (diameter > 4 cm) and can result in various symptoms and signs, including palpable mass, flank pain, urinary tract infections, spontaneous hemorrhage, and in more rare circumstances, arterial hypertension, hemorrhaging and kidney failure [7, 17, 18]. Recommendations for the clinical management of angiomyolipomas include an abdominal MRI every 1–3 years to monitor renal disease progression [19–21]. Angiomyolipomas associated with bleeding should be treated by vascular embolization and corticosteroids. For angiomyolipomas larger than 3 cm in diameter, recommendations indicate that the first-line therapy should be a mechanistic target of rapamycin (mTOR inhibitor) [20], a protein that in humans is encoded by the mTOR gene regulating cell growth, proliferation, cell motility, cell survival, protein synthesis, autophagy and transcription, and an inhibitor to prevent continued growth and bleeding [22]. Embolization and corticosteroids are recommended as first-line [21] or surgical resection is recommended as second-line therapies [20]. Because standard surgical resection can cause excessive bleeding, selective embolization has been the preferred treatment method for sparing the kidney, with surgical removal of the kidney being the last resort in cases of uncontrollable hemorrhage [7].

Most individuals who are mildly affected by TSC angiomyolipomas lead active and productive lives, but angiomyolipomas may continue to develop through the lifespan and individuals may require continuous follow-up care. There is little evidence available describing the real-world morbidity, clinical practice patterns, and health-related quality of life of either pediatric or adult patients with TSC angiomyolipomas [23, 24]. Previous research presented burden of illness results from the total TSC sample [25], a subset of the sample reporting subependymal giant cell astrocytomas (SEGAs) [26], and the physical and mental health impact on caregivers of patients with TSC [25].

The objective of this study is to describe the age stratified morbidity, health care utilization, and health-related quality of life of members of the TSC patient community with angiomyolipomas in the United States (US).

## Methods

### Study design

A cross-sectional, web-based survey was administered to TSC patients and caregivers in the US who were affiliated with social media networks of the Tuberous Sclerosis (TS) Alliance, a US-based patient advocacy organization (http://www.tsalliance.org/). The survey instrument and recruitment methodology, developed by the authors, were approved by an institutional review board. Participants provided online consent prior to completing the anonymous survey. Survey completion time was 45–60 min. Completers were eligible to receive a $25 gift card link, or donate the $25 to either the TS Alliance or National Organization for Rare Disorders.

### Study sample

Participants were recruited via e-mail and social networking web sites associated with the TS Alliance in May and June 2012. Approximately 3000 to 4000 people were members or followers of the web sites and would have been invited to participate but it is unknown how much duplication there would have been between the sites. Eligible patient respondents were 18 years of age or older, diagnosed with TSC, and able to participate in a web-based survey. Eligible caregiver respondents had to be 18 years or older, care for someone diagnosed with TSC, and able to participate in a web-based survey. The survey was completed by either a TSC patient (over 18 years) or a caregiver of a TSC patient of any age but not both. Caregivers of TSC patients with cognitive impairments were eligible to participate in the survey and provided proxy report for clinical and some demographic information.

### Survey components

Survey components included standardized patient-reported outcome measures and questions drawn from existing survey sources or developed by the study team. Self-report information related to the number of and most bothersome manifestation of TSC was gathered. Participants were asked which health care practitioners they visited in the past year. Based on their responses, they were asked follow-up questions about visits and resource use in the past 6 months or year using checklists. Resource use included tests or procedures, hospitalizations, emergency room visits, or surgical procedures in the past year. Participants who reported kidney complications were asked follow-up questions about the type of impaired kidney function and any dialysis or renal surgery such as embolization (e.g., for kidney lesions), nephrectomy (kidney removal), or kidney transplant.

Standardized patient-reported outcome measures within the survey included the 12-Item Short Form Health Survey, version 2 (SF-12) and Hamilton Depression Inventory Short Form (HDI-SF). Patients and caregivers both self-reported responses for the SF-12 and HDI-SF. The SF-12 is a widely-used, generic health status instrument measuring various aspects of health-related functioning over the preceding 4 weeks. The instrument’s eight health domains are: Physical Functioning; Role-Physical; Bodily Pain; General Health; Vitality; Social Functioning; Role-Emotional; and Mental Health. Two summary scores, the Mental Component Summary and Physical Component Summary, can be calculated with scores ranging from 0 to 100; higher scores indicating better functioning [27]. These summary scores are useful for comparing the relative burden of disease [28–32].

The HDI-SF is a 9-item measure capturing depressive symptoms over the last 2 weeks [33]. Scores for the questionnaire range from 0 to 33 with higher scores indicating more depression symptoms. Scores greater than 8.5 are indicative of mild to more severe depression which may need evaluation for clinical depression [34, 35]. Patient self-report data for depressive symptomatology are reported and compared to non-psychiatric community dwelling individuals [35]. The Work Productivity and Activity Impairment questionnaire was also included in the study but not reported in these results.

Sociodemographic questions including age, gender, race, and insurance status were also completed by respondents.

### Statistical analysis

All available data from the survey were used for the analysis; no data were imputed. Analyses were conducted on two age-based subgroups of angiomyolipoma patients: a pediatric group (≤18) and an adult group (> 18 years of age). Descriptive statistics were used to characterize the sample in terms of sociodemographic and clinical characteristics, disease experience, and health care resource utilization. Patient self-report SF-12 data were compared to normative data for patients with kidney disease (*n* = 80) [36] using Student’s t-test. All analyses were conducted using SAS version 9.2.

## Results

### Sample description, sociodemographic and clinical characteristics

Of the 676 survey respondents, 182 (27%) reported kidney complications. Seventeen percent reported a diagnosis of subependymal giant cell astrocytomas, 38% reported being diagnosed with seizures, and 53% reported being diagnosed with skin lesions. Patients were diagnosed with TSC at the mean age of 21.8 (±15.5 SD) years and the majority of patients had commercial/private health insurance (33%) or Medicaid insurance (23%). Eighty-five percent of respondents were Caucasian and 41% (*n* = 275) were caregiver respondents.

Of the total sample with kidney complications (*n* = 182), 110 (60%) reported a diagnosis of renal angiomyolipoma, of which 79 (72%) were adult patients and 31 (28%) were pediatric age patients. The demographic characteristics of the patients with angiomyolipoma are presented in Table 1 alongside characteristics for the total sample. More than half of the survey respondents in the angiomyolipoma group were caregivers. The mean age for the overall angiomyolipoma patient sample was 29 ± 17 years. The mean patient age for the pediatric group was 11 ± 5 years and 36 ± 14 for the adult group.

**Table 1:** Demographics of TSC Angiomyolipoma (AML) Patients

	TSC-AML Pediatric Patients (Age < = 18) (*N* = 31)	TSC-AML Adults Patients (Age > 18) (*N* = 79)	TSC-AML All Patients (*N* = 110)	Total TSC Sample (*N* = 676)
Caregiver Respondents, n (%)	31 (100%)	28 (35%)	59 (54%)	275 (41%)
Mean Patient Age (SD)	10.6 (4.7)	36.4 (13.6)	29.1 (16.6)	29.8 (17.7)
Median (min-max)	10.0 (2–18)	35.0 (19–73)	26.5 (2–73)	30.0 (0–83)
Health Insurance Status, n (%)				
Medicaid^a^	12 (39%)	19 (24%)	31 (28%)	157 (23%)
Medicare^b^	5 (16%)	10 (13%)	15 (14%)	109 (16%)
Medicaid + Medicare^c^	–	11 (14%)	11 (10%)	102 (15%)
Military^d^	1 (3%)	7 (9%)	8 (7%)	53 (8%)
Commercial/private only	13 (42%)	24 (30%)	37 (34%)	223 (33%)
Other/none	–	8 (10%)	8 (7%)	32 (5%)

^a^Includes Medicaid only or Medicaid plus private insurance

^b^Includes Medicare only or Medicare plus private insurance

^c^Includes Medicaid plus Medicare or Medicaid plus Medicare plus private insurance

^d^Includes VA/military/Tricare or Medicaid/Medicare/VA/military/Tricare

*AML* Angiomyolipoma, *SD* standard deviation, *TSC* Tuberous sclerosis complex

Eighty-four percent of the pediatric group and 76% of the adult group reported lesions on both kidneys. Of the patients experiencing involvement of only one kidney, 60% of the pediatric group and 21% of the adult group reported having multiple tumors within the affected kidney. Thirteen percent of the pediatric group reported impaired kidney function, 13% reported chronic kidney disease, and 10% reported hypertension. Twenty-nine percent of the adult group reported kidney failure, while nearly half (49%) reported impaired kidney function and 38% chronic kidney disease.

All of the pediatric group and 77% of the adult group reported having three or more other TSC manifestations besides angiomyolipoma. In the pediatric group, the most prevalent manifestations were: seizures and skin lesions (100%), cognitive impairments (84%), and subependymal nodules/cerebral tumors (77%). Respondents for the pediatric group reported kidney complications (32%), seizures (22%), and cognitive complications (12%) as their most bothersome tuberous sclerosis complex manifestation. For the adult group, the most prevalent manifestations reported were: skin lesions (75%), seizures (57%), or eye problems (48%). Over one-third of the adult group reported kidney complications as their most bothersome TSC manifestation.

### Medical practice patterns / health care utilization

Tables 2 and 3 describe the healthcare utilization for TSC reported by the angiomyolipoma patient groups over the past year. A small proportion of pediatric patients progressed to the point of receiving embolization for a kidney lesion (6.5%), or nephrectomy (6.5%) and none reported having dialysis (0%) (Table 2). Renal procedures were undertaken more frequently among the adult group: 29% reported receiving embolization for a kidney lesion, 17% reported having a kidney transplant, and 15% a nephrectomy.

**Table 2:** Kidney-related Outcomes

Characteristics	TSC-AML Patient Age < = 18 (*N* = 31)	TSC-AML Patient Age > 18 (*N* = 79)	Total TSC-AML Patients (*N* = 110)
Embolization (e.g., for kidney lesions)	2 (7%)	23 (29%)	25 (23%)
Nephrectomy (kidney removal)	2 (7%)	12 (15%)	14 (13%)
Kidney transplant	0 (0%)	13 (17%)	13 (12%)
Had impaired kidney function	4 (13%)	39 (49%)	43 (39%)
Had high blood pressure/hypertension	3 (10%)	35 (44%)	38 (35%)
Had chronic kidney disease	4 (13%)	30 (38%)	34 (31%)
Had kidney failure	0 (0%)	23 (29%)	23 (21%)
On dialysis	0 (0%)	17 (22%)	17 (16%)
AML tumor involvement			
In both kidneys	26 (84%)	60 (76%)	86 (78%)
Only in one kidney	5 (16%)	19 (24%)	24 (22%)
Multiple tumors	3 (60%)	4 (21%)	7 (29%)
Only one tumor	2 (40%)	15 (79%)	17 (71%)

*AML* Angiomyolipoma, *TSC* Tuberous sclerosis complex

**Table 3:** Healthcare Utilization for TSC Over the Past Year

Health Care Use	TSC-AML Pediatric Patients (Age < = 18) (*N* = 31)	TSC-AML Adult Patients (Age > 18) (*N* = 79)	TSC-AML All Patients (*N* = 110)
Physician visits in past year, n (%)	31 (100%)	78 (98.7%)	109 (99.1%)
Mean (SD) no. visits/year	34.7 (344)	28.6 (22.3)	30.4 (26.3)
Median (IQR) no. visits/year	24.0 (21.0)	24.0 (21.0)	24.0 (22.5)
Procedures/tests in past year, n (%)	31 (100%)	78 (99%)	109 (99%)
Mean (SD) no. tests/year	11.9 (7.9)	11.3 (8.3)	11.5 (8.1)
Median (IQR) no. tests/year	11.0 (13.0)	9.0 (9.0)	10.0 (9.0)
Hospitalizations in past year, n (%)	13 (42%)	35 (44%)	48 (44%)
# times admitted in past year			
Mean (SD) no. hospitalizations/year	2.4 (1.9)	2.4 (1.4)	2.4 (1.5)
Median (IQR) no. hospitalizations/year	2.0 (2.0)	2.0 (2.0)	2.0 (2.0)
Days in hospital			
Mean (SD) no. days/hospitalization	5.1 (3.7)	5.6 (3.2)	5.5 (3.3)
Median (IQR) no. days/hospitalization	5.0 (5.0)	5.0 (5.0)	5.0 (4.5)
Emergency room visits in past year, n (%)	14 (45%)	29 (37%)	43 (39%)
# times in ER in past year			
Mean (SD) no. visits/year	2.6 (1.7)	2.4 (1.4)	2.4 (1.5)
Median (IQR) no. visits/year	2.0 (2.0)	2.0 (2.0)	2.0 (2.0)

*AML* Angiomyolipoma, *IQR* interquartile range, *SD* standard deviation, *TSC* Tuberous sclerosis complex

The entire pediatric group (100%) and nearly the entire adult group (99%) reported visiting a physician and undergoing medical procedures or tests in the past year for TSC (Table 3). Similar proportions of pediatric and adult patients reported being admitted to the hospital for any TSC reason (42% and 44%). A higher proportion of pediatric patients reported visiting an emergency room for their TSC compared to adult patients (45% vs. 37%). The median number of physician visits for TSC-related concerns was 24 in both age groups while the median number of procedures or tests for TSC was 11 for the pediatric group and 9 for the adult group.

Of the 31 pediatric patients, caregivers responded that 12 (39%) had undergone brain surgery to place or replace a shunt and 12 (39%) had undergone laser surgery to remove skin lesions. Among the 79 adult patients, 20 (25%) reported laser surgery, 9 (11%) had brain surgery to replace a shunt, 8 (10%) had brain surgery for SEGA, and 7 (9%) had brain surgery for epilepsy.

The majority of patients reported taking two or more medications for TSC. The most prescribed classes of medications for pediatric patients were anti-epileptic (93%) and sleep (29%) medications. For the adult patient group, antidepressants/anti-anxiety/anti-psychotics (52%) were reported as most common, followed by anti-epileptic (45%) and sleep (29%) medications.

The most frequent tests and procedures for pediatric patients over the previous year were blood work (87%), magnetic resonance imaging (MRI) (84%), ultrasounds (74%), and TSC-related eye exams (55%). For the adult patients, the most common test was blood work (72%) followed by MRI and CT scan (56%) and ultrasound (53%).

Seventy-five percent of the adult group lives more than 100 miles from a tuberous sclerosis complex–specific clinic whereas 48% of the pediatric group lives that far away, but 41% of the adult group and 45% of the pediatric group reported receiving most of their care from a tuberous sclerosis complex clinic. The pediatric group spent a median of 15 h over the past year traveling to and from medical appointments for tuberous sclerosis complex care while the adult group spent 20 h.

### Health-related quality of life burden

A total of 51 adult patients completed the SF-12 (Table 4) and HDI-SF, which were used to estimate the effect of TSC on their health-related quality of life. Caregiver respondents did not complete the SF-12 as proxy respondents for their patient.

**Table 4:** HRQL: Angiomyolipoma vs. US-based Patient Norms

SF-12v2 Scores	TSC-AML Adult Patients (Age > 18) (*N* = 51) Mean (SD)	US Norms for Kidney Disease Patients^a^ (*N* = 80) Mean (SD)	*P* value^b^
Physical Component Summary (PCS)	39.6 (9.0)	37.9 (11.2)	0.185
Mental Component Summary (MCS)	42.5 (8.0)	45.2 (10.1)	0.0186

^a^Mean age for kidney disease norm data 56.8 years, 57% female. Reference: Ware et al. 2009 [36]

^b^Significance level of Student’s t-test angiomyolipoma vs. kidney disease

*AML* Angiomyolipoma, *HRQL* health-related quality of life, *MCS* mental component summary, *PCS* physical component summary, *SD* standard deviation, *SF-12v2* Short form 12-item survey, version 2.0, *TSC* Tuberous sclerosis complex, *US* United States

There was no significant difference between the TSC angiomyolipoma population and the non-TSC kidney disease populations on the Physical Component Summary score (Physical Component Summary score: 39.6 vs. 37.9, *p* = 0.185). Adult patients reported significantly lower (worse) mean Mental Component Summary scores compared to US-based norms for kidney disease patients (Mental Component Summary score: 42.5 vs. 45.2, *p* < 0.05; mean age 56.8 years, 57% female).

Adult patients reported a mean depressive symptom score of 9.4, above the 8.5 cut-off indicative of presence of depressive symptoms. Compared to non-psychiatric community dwelling individuals, adult angiomyolipoma patients experience significantly higher depressive symptoms (9.4 ± 6.0 vs. 5.1 ± 3.6, *p* < 0.0001).

## Discussion

This survey describes the heterogeneity and age-related severity of illness experienced by TSC patients with angiomyolipomas, as well as their healthcare resource utilization, and health-related quality of life burden. Renal disease poses a significant burden on patients with TSC and renal failure has previously been identified as a leading cause of death in TSC [12]. Due to the heterogeneity of TSC and potential delays in diagnosis and the increasing age-related morbidity, it is important to understand the overall burden of angiomyolipomas on patients with TSC.

Clinical differences were found between the pediatric and adult TSC angiomyolipoma groups with adult patients reporting kidney impairment at a much higher rate and higher age of diagnosis of TSC. The pediatric group reported a greater number of other TSC manifestations in addition to angiomyolipomas compared to the adult group. Interestingly though, a similar percentage of the adult and pediatric groups reported that kidney complications were the most bothersome manifestations. Also, similar health care utilization patterns were found between the pediatric and adult group even though renal complications and angiomyolipomas are recognized to worsen with patient age. Comparing the TSC-related angiomyolipoma subgroup with the TSC-related subependymal giant cell astrocytoma (TSC-SEGA) subgroup from this study [26] showed similar numbers of patients reporting physician visits, procedures, hospitalizations, and ER visits.

As a result of the high burden of illness over the life span, angiomyolipoma patients require ongoing clinical care resulting in significant utilization of health care resources including numerous health care provider visits, tests, and surgical procedures. While treatment approaches largely focus on mitigating the risk of bleeding presented by larger angiomyolipomata, renal involvement in TSC (including the presence of kidney cysts and smaller angiomyolipomas) is also associated with deterioration of kidney function [7–11]. Following TSC diagnosis, consensus recommendations call for regular abdominal imaging as well as assessment of kidney function via blood tests and evaluation of blood pressure at least annually [20, 21].

The prolonged burden of TSC presents significant challenges related to education, professional and family life for both patients and caregivers. TSC-related angiomyolipomas are associated with significant health-related quality of life burden in both the mental health and physical domains. TSC-related angiomyolipoma patients scored statistically significantly worse than normative kidney disease patients on the MCS. A study by Lacson and colleagues showed a clinically meaningful difference in the MCS from the SF-12 to be 1.7 points based on hospitalizations for long-term dialysis patients [37]. Therefore, the difference found in this study may be clinically meaningful, demonstrating that the current sample has mental well-being that is more affected than the normative comparison group, which includes patients who experience a significant burden of acute and chronic illness. Eriksson and colleagues (2017) present a study assessing HRQL across all stages of autosomal dominant polycystic kidney disease (ADPKD): chronic kidney disease, dialysis, and transplant. PCS scores for these patients ranged from 34.9 for dialysis patients to 51.2 for early chronic kidney disease patients [38]. MCS scores ranged from 47.0 for dialysis patients to 52.7 for kidney transplant patients. PCS and MCS scores in the TSC patients fall within both ranges suggesting their HRQL burden is similar to patients in Eriksson’s study.

Compared to the TSC-SEGA subgroup [26] the subgroup with TSC-related angiomyolipomas reported similar physical domain scores but better mental health scores. When the TSC-related angiomyolipoma subgroup was compared to the caregiver sample [25] the two groups demonstrated similar MCS scores but caregivers reported better PCS scores. Although these analyses and results are exploratory, they do help us understand the extent of burden in these TSC-related angiomyolipoma patients.

Since the study was based on a convenience sample of TSC patients and their caregivers, the generalizability of these results is limited. The anonymous web-based data collection process prevented clinical verification of the self-report data. While some manifestations reported by respondents in this sample, such as SEGA and skin manifestations, are within prevalence ranges reported in the literature, the 17% prevalence of angiomyolipomas in this sample is much lower than reported in other studies [8, 10, 16, 39] but similar to the prevalence reported in a retrospective, longitudinal study from the UK [24]. Some studies are inconclusive as to the actual prevalence of angiomyolipomas. In a longitudinal renal surveillance study by Ewalt and colleagues based in England, of the 60 children identified with TSC, the authors found that 55% of the children (mean age of 6.9 years) had a renal lesion upon initial observation, yet after a decade of follow-up observation, a cumulative incidence of 80% was reported in this cohort [39]. In another prospective study by O’Callaghan et al. (2004) to establish the prevalence of renal angiomyolipoma in the south of England, 13% of TSC patients had a history of renal symptoms in the previous year, yet 69% reported having renal angiomyolipomas [10]. Most angiomyolipoma patients in this study reported bilateral kidney impairment, with differences between pediatric and adult groups. The proportion of respondents reporting kidney embolization and transplant was higher in this sample than other published data [10, 39]. As one might expect with chronic conditions, more adult patients reported treatment for chronic kidney conditions than did pediatric patients: 3–4 times as many adult patients compared to pediatric patients reported an embolization, nephrectomy, transplant, or were on dialysis. In our sample, the prevalence of embolization, nephrectomy and kidney transplant was higher than in some studies [40], but also lower than others [41]. In a nationwide survey of adult and pediatric dialysis centers with 65 TSC patients, over one quarter of those patients reported having had a functional transplant [42]. Perhaps with new treatment options available [43] and becoming part of treatment guidelines, the proportion of pediatric patients eventually requiring a functional transplant may decrease.

A few epidemiological studies have identified lower prevalence of angiomyolipomas and indicate that there may be underreporting of symptoms or complications because of problems eliciting accurate histories from patients with learning difficulties and from their caregivers [10]. Another TSC patient questionnaire-based study conducted by van Eeghen et al. (2012) reported self-report data from TSC patients, even given low response rates (25%), to characterize sleep patterns and possible association with mental health impacts [44].

A limitation of these results is that they may reflect an unrepresentative sample due to self-selection. Participants were recruited through social networking internet sites associated with the TS Alliance and a few additional patient advocacy websites. Only people registered with or following these sites would be alerted of the study. These respondents may comprise a group of people who are more involved and interested in TSC and advocating for change in available treatments or healthcare for TSC. Additionally, another limitation may be due to recall bias. Respondents were asked to recall treatment received, costs incurred, or time spent on TSC over a period of time ranging from the past 6 months to a year. While cognitive impairment may affect patients with TSC potentially calling into question some recall results, this sample seemed to have less neurological impairment due to their TSC based on the relatively high level of education reported by both caregiver and patient respondents. The accuracy of self-reported healthcare resource utilization has been previously studied and findings indicate that self-report of health care resource use may be similar to data abstracted from medical records over varying recall periods for patients with higher performance levels or better health status [24, 45, 46] and over-reported in patients with greater number of health care visits [47] although this relationship was not found always to be true [48].

## Conclusion

Renal angiomyolipomas are a common manifestation in patients with tuberous sclerosis complex. Patients with renal angiomyolipomas report continuing clinical care including numerous visits, tests, and surgical procedures. Mental health-related quality of life is also affected for patients with renal angiomyolipomas. The information presented here on the clinical and health-related quality of life burden experienced by pediatric and adult patients with angiomyolipomas due to tuberous sclerosis complex should help further inform health care providers about the burden faced by patients and family caregivers. This cross-sectional, convenience sample of angiomyolipoma patients could be complemented by future longitudinal research combining patient/caregiver input with data provided by physicians and health care providers. Conducting additional research after implementation of the 2012 recommendations for diagnosis and treatment might result in differences in prevalence and treatment patterns [20, 49]. Finally, this research should be replicated in other geographic locations, where patients may experience access to different health care systems and different diagnosis and treatment patterns.

## Abbreviations

AML: Angiomyolipoma
CT: Computed tomography
MRI: Magnetic resonance imaging
mTOR: Mechanistic target of rapamycin (formerly known as mammalian target of rapamycin)
SD: Standard deviation
SEGA: Subependymal giant cell astrocytoma
SF-12: 12-item short form health survey, version 2
TOSCA: Tuberous sclerosis registry to increase disease awareness
TS: Tuberous sclerosis
TSC: Tuberous sclerosis complex
US: United States
